# Diketopiperazine Gels: New Horizons from the Self-Assembly of Cyclic Dipeptides

**DOI:** 10.3390/molecules26113376

**Published:** 2021-06-03

**Authors:** Marco Scarel, Silvia Marchesan

**Affiliations:** 1 Chemical and Pharmaceutical Sciences Department, University of Trieste, Via Giorgieri 1, 34127 Trieste, Italy; marco.scarel2@studenti.units.it; 2 National Interuniversity Consortium of Materials Science and Technology (INSTM), University of Trieste, 34127 Trieste, Italy

**Keywords:** cyclopeptides, diketopiperazines, gels, hydrogels, peptides, amino acids, self-assembly, supramolecular, nanomaterials, microwave

## Abstract

Cyclodipeptides (CDPs) or 2,5-diketopiperazines (DKPs) can exert a variety of biological activities and display pronounced resistance against enzymatic hydrolysis as well as a propensity towards self-assembly into gels, relative to the linear-dipeptide counterparts. They have attracted great interest in a variety of fields spanning from functional materials to drug discovery. This concise review will analyze the latest advancements in their synthesis, self-assembly into gels, and their more innovative applications.

## 1. Introduction

Gels based on self-assembling biomolecules have become a popular research topic [1]. Amongst their attractive features, there is also good potential as greener alternatives to traditional synthetic polymers. They do not persist in the environment and are not derived from oil-industry raw materials. In particular, self-assembling short-peptides represent cheap building blocks for large-scale production, and with overall promising properties in terms of bioactivity [2] and biocompatibility to achieve functional materials [3,4]. The large chemical diversity they provide ensures continuous advancements, and even considering just the publications in the last couple of years, numerous regard self-assembling tripeptides [5,6,7,8,9,10,11,12,13,14,15] and dipeptides [16,17,18,19,20,21,22,23,24,25]. Furthermore, the study of such simple systems could advance the understanding of prebiotic chemistry, relative to the potential role of peptide-based organized structures in the emergence of catalytic activity [26].

**Figure 1:**
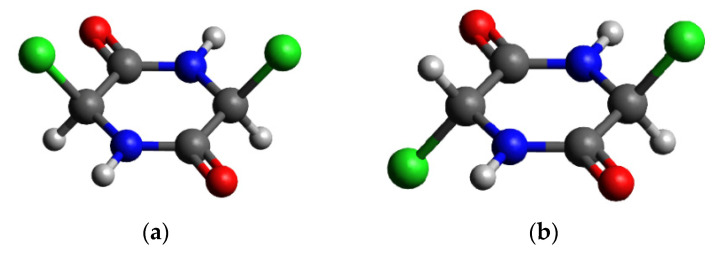
3D Chemical structure of generic cyclodipeptides (CDPs) obtained from the cyclization of L-homochiral (**a**) or D,L-heterochiral (**b**) dipeptides. Side chains are represented as a generic green sphere and the rest of the atom types are represented using the standard CPK model.

Linear dipeptides can easily cyclize into 2,5-diketopiperazines (DKPs) or cyclodipeptides (CDPs), which are widely reported as self-assembling building blocks in virtue of their enhanced rigidity and hydrogen-bonding into defined networks, both of which can be advantageous for self-organization into gels [27]. The orientation of the amino acid lateral chains (Figure 1) could be on the same side of the ring plane for the homochiral CDPs (LL/DD), or on the opposite side for the heterochiral stereoisomers (LD or DL). The ring structure increases the resistance against proteolytic degradation compared to linear peptides and allows them to cross the blood-brain barrier, both of which are important aspects, for instance, for the development of neuroprotective agents for oral administration [28]. CDPs are indeed attracting interest as drug carriers, thanks to their demonstrated ability to cross membranes and penetrate cells [29]. Their aggregates can display fluorescence [30], and they are widely studied for their diverse biological activities [31,32,33,34,35,36,37,38], which make them promising for biomedical applications, as recently reviewed [39]. Further, they are being investigated also for optoelectronic [40] and catalytic [41] applications, as biodegradable and environmentally-friendly substitutes of more traditional options.

All these features make CDPs an attractive choice as biocompatible, green, functionalizable, and cheap building blocks for smart materials with a wide scope of applications from industry to medicine. The basic design is very simple: two amino acids bound together in a cyclic structure with a defined stereochemistry (i.e., LL, LD/DL, or DD) and the possibility to further derivatize the side chains [42,43,44,45,46] of selected amino acids [47,48,49,50], as widely applied in nature [51,52,53,54,55,56].

CDPs are synthesized by microorganisms [57,58], they are found in food as tasting agents, and can be the product of fermentation processes that are particularly relevant to the food and beverage industry [59]. A dipeptide is a relatively simple structure, and it is easy to synthesize for modern organisms, thanks to the complex molecular machinery provided by ribosomes and enzymes. CDPs can also form spontaneously without enzymes, and therefore interest in their role as prebiotic-chemistry building blocks is growing [60]. The nature of the side chains of CDPs is not only involved in the self-assembly process but they could also provide catalytic functional groups. Thus, materials obtained by these molecules could be versatile [41]. There is growing interest in CDPs, and recent reviews exist that discuss their bioactivity [58,61,62], supramolecular organization [31,40], and use as scaffolds for drug discovery [52,63,64,65,66].

Therefore, this concise review will focus on gels obtained through the self-assembly of CDPs formed by unprotected and underivatized amino acids. Hydrogels and organogels are the two types of materials that are mostly studied; xerogels are obtained upon drying gels, and often their purpose is the characterization of the system whenever it is not possible to use a hydrogel, for example in transmission-electron microscopy (TEM) and infrared (IR) spectroscopy techniques. Conversely, studies of aerogels and ionogels based on CDPs are still scarce [67], therefore these two types of materials will not be discussed.

## 2. Synthesis of CDPs

### 2.1. Organic Synthesis of CDPs in Solution

**Scheme 1:**
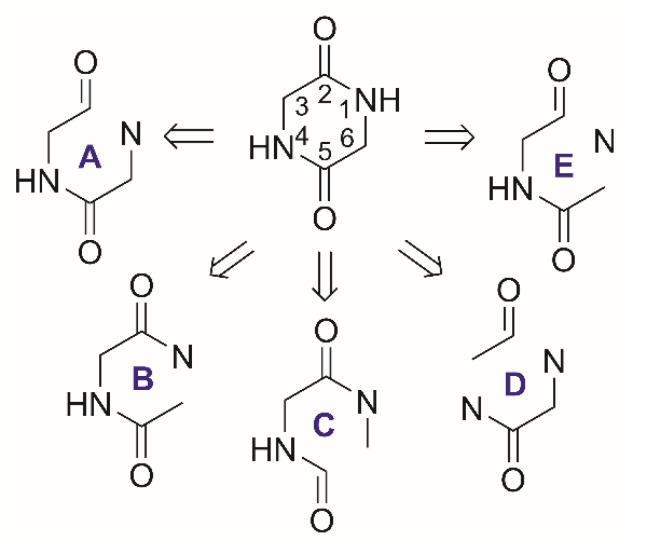
Retrosynthetic analysis reveals five disconnections (A–E) to yield the CDP ring [63].

Retrosynthetic analysis of the CDP ring (Scheme 1) reveals several possible disconnections with routes A–B being far more popular than tandem cyclizations (D–E). The most popular route, which can be easily performed also in water or solvent-free through green approaches, goes through the disconnection of the amide bond (Scheme 1, A), and several methods to cyclize dipeptides for this approach are described further below. An aza-Wittig cyclization will also go through disconnection A, as will do Ugi chemistry using an isonitrile, an acid, an aldehyde, and an amine. The C-N disconnection (Scheme 1, B) is another quite popular alternative that involves intramolecular N-alkylation. Also, in this case, 4-component Ugi-type chemistry can be employed, in tandem with an Aza-Michael. Alternatively, the use of a Diels-Alder allows to achieve CDPs with a high level of structural complexity. The C-C disconnection (Scheme 1**,** C) often involves enolate acylation, albeit radical routes were also described to attain the C-C cyclization [63]. Despite the various synthetic routes available, the search is still active in this area with new developments pertaining to CDP synthesis appearing also in the most recent literature [68,69,70,71,72,73,74,75,76].

### 2.2. Organic Synthesis of CDPs in Solid Phase

**Scheme 2:**
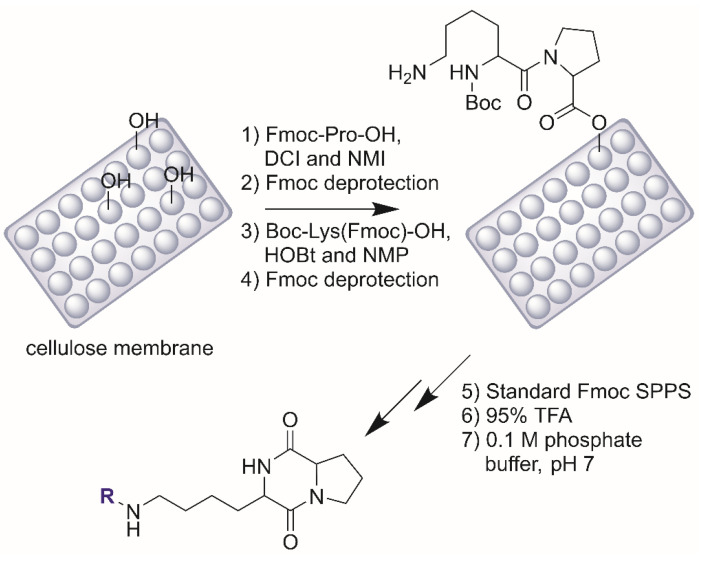
CDP-array synthesis on cellulose membranes via SPOT method for rapid screening [80]. Boc = tert*-butyloxycarbonyl;* DIC = *N,N′*-diisopropylcarbodiimide; Fmoc = fluorenylmethoxycarbonyl; HOBt = Hydroxybenzotriazole; Lys = lysine; NMI = *N*-methylimidazole; NMP = *N*-methyl-2-pyrrolidone; Pro = proline; SPPS = solid-phase peptide synthesis; TFA = trifluoroacetic acid.

Solid-phase synthesis of CDPs [77,78] offers the possibility to rapidly gain access to a plethora of structurally complex products [79]. Solid-phase methods can be very convenient for the rapid screening of arrays of libraries obtained through the parallel synthesis of CDPs, for instance via the SPOT method on cellulose membranes (Scheme 2) [80]. This type of approach to preparing CDPs is very convenient on a small scale when the priority is time. Conversely, it is rather costly and poses challenges for scale-up, so it will not be described further here, since several convenient liquid-phase alternatives are available.

### 2.3. Dipeptide Cyclization to CDPs

#### 2.3.1. Liquid-Phase Cyclization

Dipeptide cyclization is certainly the most widely used approach and can be performed starting with a C-terminally protected dipeptide, both in organic solvent and water. The reaction is an aminolysis, therefore the N-terminus must be deprotected either before the reaction or during the cyclization in a one-pot fashion. The C-terminal protecting group is usually a methyl ester so that the N-terminal amine acts as the nucleophile and the methoxy functionality is the leaving group. For example, the cyclization of aspartame (l-Asp-l-Phe-OMe) was studied in an organic solvent. The reaction was performed in dimethylsulfoxide (DMSO), by stirring the solution for 8 h at 80 °C with a final yield after workup of 88% [81]. Dipeptides featuring a methoxy group at the C-terminus could also spontaneously cyclize in water via aminolytic reaction [82].

#### 2.3.2. Microwave-Assisted Cyclization

**Scheme 3:**
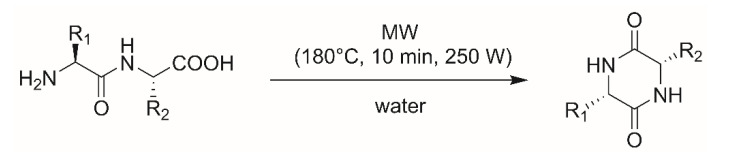
Microwave-assisted cyclization of terminally unprotected dipeptides in water occurs in nearly quantitative yield with no need for workups [86]. R_1_ = benzyl; R_2_ = benzyl; *iso*-propyl.

Microwave-assisted cyclization in water is an interesting technique that allows one to make CDPs in high-yield and within a short time. In the case of hydrophobic CDPs, the workup is straightforward due to the precipitation of the product. With this technique, it is possible to perform a greener synthesis of CDPs because more steps are merged in one, avoiding expensive workups and petroleum-derived solvents. Microwave heating was studied in many different solvents for dipeptide methyl esters and only in water it is possible to reach high yields [83]. It is possible to remove the Boc (*tert*-butyloxycarbonyl) protecting group through microwave heating in water when amino acids or peptides display an unprotected C-terminus [84]. However, N-Boc deprotection and cyclization can also occur in one-pot, as demonstrated on C-terminal *tert*-butyl and methyl esters [85]. Recently, the green synthesis of CDPs was obtained in high-to-quantitative yield by microwave-assisted cyclization in water (Scheme 3), also without the methyl ester at the C-terminus, and with no need for work-ups for hydrophobic CDPs that precipitate as white solids, leaving any unreacted dipeptide in solution [86].

#### 2.3.3. Cyclization Using Vapor Deposition

A peculiar case of dipeptide cyclization was described using vapor deposition [87]. Diphenylalanine (Phe-Phe) was heated at 220 °C and thus evaporated in a vacuum chamber. When the peptide reached a cooler surface (i.e., 80 °C), it cyclized and formed nanotubes. The stacking interactions between the aromatic rings of the side chains were the key for inter-molecular recognition and templating of the cyclization to CDPs, as well as the growth of their self-assembled nanotubes.

#### 2.3.4. Cyclization in the Solid State

CDPs can be synthesized also in the solid state, through the heating of a powder sample [88,89]. The appropriate reaction temperature depends on the nature of the peptide sequence. For example, Gly-Gly needs 230 °C, while Phe-Phe 147 °C [90] or 125 °C [91], suggesting a correlation between the reaction minimum temperature and the amino acid side-chain steric hindrance. Cyclization indeed requires molecular mobility, which is governed by the nature of the side chains in the solid state. The melting points of Gly-Gly and Phe-Phe are respectively 262–264 °C and 288–290 °C, thus confirming that the reaction occurred in a solid state and probably above the glass transition temperature (*T*_g_). The latter depends on the sample preparation when manufactured; from that point onwards, CDP molecules do not maintain random conformations and, instead, they start to reorganize in the solid structure, since rotations and translations are possible [90].

### 2.4. Enzymes and Biotechnological Tools for CDP Synthesis

**Figure 2:**
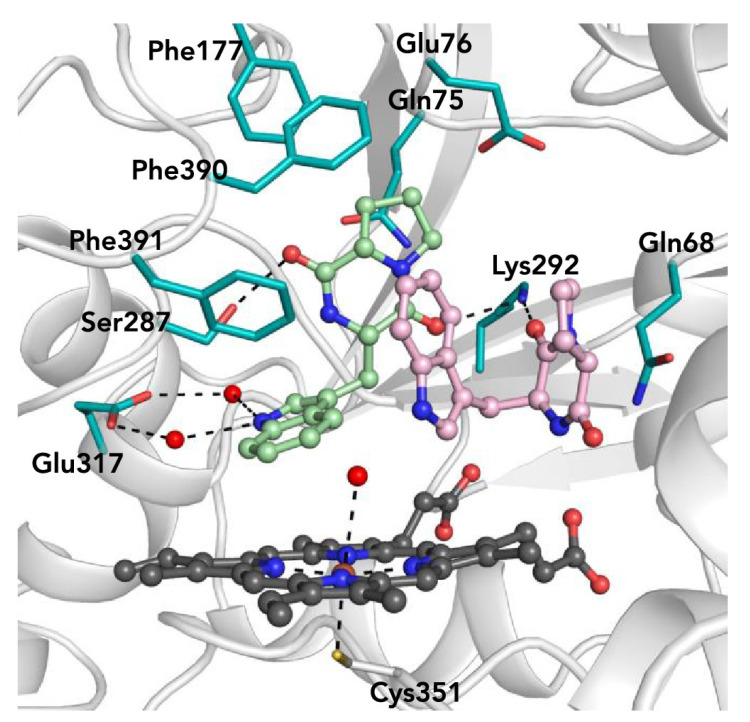
Active site of cytochrome P450 NzeB showing the concave substrate (pink) bound in the “cyclization site”, and the extended substrate (green) bound in the “dimerization site”. Reproduced with permission from [96], copyright © 2020, American Chemical Society.

Finally, biotechnological tools are also being developed to prepare CDPs. To this end, cyclodipeptide synthases were recently reported to catalyze the cyclization of aminoacylated-tRNA substrates [92,93]. Installation of C-C double bonds to attain dehydrogenated CDPs can be achieved through oxidase-mediated catalysis [94], while N-alkylation is catalyzed by methyltransferases [95]. Interestingly, a cytochrome P450 has been identified that can catalyze both the CDP dimerization and cyclization towards bioactive CDP-derived natural products with interesting bioactivities (Figure 2) [96]. Mutagenesis of other cytochrome P450s gave access to catalysts for CDP synthesis with good regio- and stereo-specificity, and chemical versatility [97]. Whole-cell biocatalysts were used to attain heterodimeric CDPs, with *Mycobacterium smegmatis* demonstrating to be a more robust and efficient organism, than the commonly used *Escherichia coli* or *Streptomyces* systems [98]. The large variety of reactions catalyzed by cytochrome P450s to synthesize and modify CDPs is gaining momentum and has just been reviewed [99].

## 3. Self-Assembly of CDPs into Gels

### 3.1. Gels from Unprotected-Dipeptide Derived CDPs

#### 3.1.1. Non-Covalent Interactions Responsible for Gelation

Supramolecular gels are very interesting because of their self-organized structure via non-covalent interactions. This feature allows one to make dynamic materials with self-healing properties and thermal reversibility avoiding any by-products, which are net advantages over classic covalent interactions. The driving force for gel transition is non-covalent, therefore it is important to evaluate the CDP structure in terms of available interactions, such as hydrogen bonding, π-π stacking, CH-π, or dipole-dipole interactions, etc. Numerous examples of gels formed by unprotected-dipeptide derived CDPs have been reported (Table 1), although the number of studies involving modified CDPs is far greater, as discussed in Section 3.2.

The role of hydrogen-bond interactions between CDP rings was studied on organogels made by cyclo(Leu-Leu) [108]. This compound is a very interesting organogelator, which self-assembles into aromatic solvents, such as benzene, xylene, toluene, and ethylbenzene, whilst not in aliphatic solvents. Surprisingly, it was noted that a small amount of water addition, not only improved the kinetics of the gelation process (that was accelerated from two days to six hours in toluene) but also allowed to attain organogels with aliphatic hydrocarbons, such as cyclohexane, hexane, and heptane. The cases of the latter three are very interesting because, in the absence of any functional group in the solvent molecules, Van der Waals forces are the only possible interactions available [108]. Hydrogen bonds play an important scaffolding role in the assemblies, while van der Waals forces are typically the driving force in the initial stages to generate the hydrophobic core of the supramolecular structures [109].

**Figure 3:**
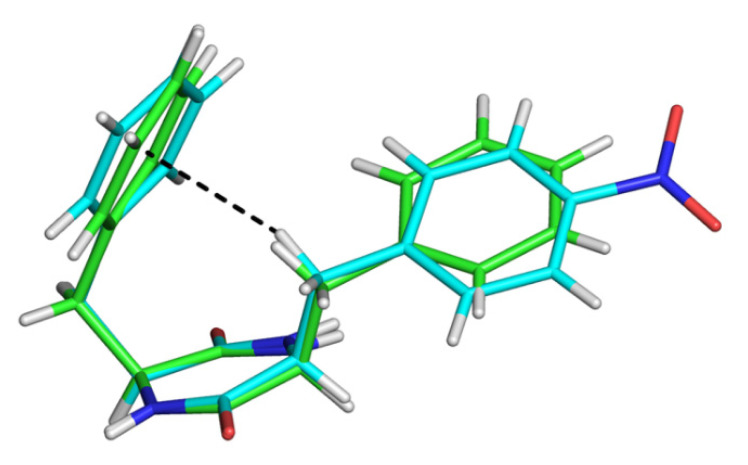
Superimposition of the single-crystal XRD structures of cyclo(Phe-Phe) (green carbon atoms) and cyclo(p-nitro-Phe-Phe) (cyan carbon atoms) reveal the same conformation with an intramolecular CH-π interaction (dashed line). Nitrogen, oxygen, and hydrogen atoms are colored in blue, red, and white, respectively. Reproduced with permission from [86], Copyright © 2019, Thieme Verlag, Germany.

The nature of the side chains of CDPs plays a role in the final structure of the supramolecular system. The presence of different functional groups allows one to expand the non-covalent interaction toolkit of CDPs. Further, they have a great influence on the solubility of CDPs in one particular solvent. Short and hydrophilic side chains are not suitable for hydrogels as seen in the case of the aspartame CDP [82]. Phenylalanine, tyrosine, tryptophan, and histidine are the four natural aromatic amino acids; the latter is also a base and, thus, it is easily ionized at different pH values. Phe, Tyr, and Trp are hydrophobic, and they could be a good choice for a potential hydrogelator. All three derived CDPs were studied as gelling agents in water and many organic solvents [104]. In particular, cyclo(Tyr-Tyr) is a robust hydrogelator and it is also an organogelator for alcohol-based solvents. Cyclo(Phe-Phe) is an organogelator, whilst cyclo(Trp-Trp) showed a weak gelling ability only in chloroform. Conversely, another study reported cyclo(Tyr-Trp) as a robust hydrogelator [105]. All these findings raise the question about the possible driving force embodied in non-covalent interactions of aromatic moieties, such as π-π stacking and CH-π interactions. Recently, a study about cyclo(Phe-Phe) and cyclo(*p*-nitro-Phe-Phe) highlighted the role of this kind of interaction with the aid of single-crystal XRD data [86]. Both CDPs displayed the same conformation in the crystal state with an intramolecular CH-π interaction (Figure 3). However, the structure of cyclo(Phe-Phe) showed intermolecular π-π stacking and intra-intermolecular CH-π interactions. Surprisingly, the introduction of the nitro group did not affect the molecular conformation, rather, it resulted in the rigid translation of the molecule into another position, thus creating a new crystal packing, with loss of an intermolecular CH-π interaction. Nevertheless, cyclo(*p*-nitro-Phe-Phe) demonstrated to be a better hydrogelator than cyclo(Phe-Phe), thus the different electronic density between the two aromatic rings could play an important role.

#### 3.1.2. Spectroscopic Characterization Methods to Monitor Gelation

The CDP basic scaffold is a six-membered ring with two amide groups. N-H and C=O can engage in hydrogen bonding. Fourier-transformed infrared (FT-IR) spectroscopy is a good technique to observe if there are any hydrogen-bond interactions. Generally, the C=O stretching signal for an amide group falls around 1690 cm^−1^, while the N-H stretching and bending signals appear approximately at 3400 and 1500 cm^−1^, respectively. As a result of the transition from solution to gel, it is possible to observe a shift of these signals because of the hydrogen-bonding interaction. For instance, FT-IR of cyclo(Val-Leu) in chloroform solution shows a shift of 1690 to 1640 cm^−1^ for the C=O group, and from 3400 to 3320 cm^−1^ for the N-H group, as a result of gelation [102]. FT-IR of cyclo(Glu-Lys) in DMSO before and after transition also shows a more complex situation. The C=O signal is shifted from 1687–1682 cm^−1^ to 1670–1677 cm^−1^, the N-H stretching from 3515–3500 cm^−1^ to 3320–3200 cm^−1^, and the N-H bending from 1500–1510 cm^−1^ to 1520–1538 cm^−1^ [107]. Stretching signals are shifted towards lower wavenumbers when hydrogen bonding occurs.

^1.^ H-NMR is another useful spectroscopic technique to study hydrogen bonding in gels. Amide signals are shifted downfield when this interaction occurs [100]. It is not possible to measure signals from a gelling agent in a rigid packing because of the long correlation time needed. However, it is possible to study small aggregates in a solution that are representatives of the material. ^1^H-NMR spectra of cyclo(Tyr-Lys) in DMF-*d_7_* showed the situation in solution at 60 °C of pre-aggregated gelators that gelled at room temperature [106]. Amide signals were broadened and shifted from 7.78 (at 65 °C) to 8.10 ppm (at 25 °C) as a result of hydrogen bonding.

#### 3.1.3. Oscillatory Rheometry to Monitor Gelation

Rheometry is the technique of election to quantify the viscoelastic properties pertaining to gels and their precursor solutions. When the elastic or storage modulus (G’) is significantly higher than the viscous or loss modulus (G’’), the system is defined as a gel. With this technique, it is possible also to study the thermo-reversibility and self-healing properties of these systems, as well as the shear-thinning behavior that is desirable for injectability. For instance, cyclo(Phe-Xaa) (Xaa = Gly, Ser, Cys, Glu, His, Lys) CDPs were characterized by rheometry, and all the soft materials displayed thermo-reversibility [103]. All these compounds formed stable hydrogels at 4 wt%. Cyclo(Phe-Cys) was found to be the best hydrogelator of the series with as little as 0.25 wt% needed for gelation, and a remarkably high gel-sol transition temperature of 100 °C when formulated at 1 wt%. Interestingly, despite the fact that serine is isoelectronic with cysteine, cyclo(Phe-Ser) was the worst gelling agent of this series with a gel-sol transition temperature of 12 °C also with high loading (4 wt%). All these compounds, except for cyclo(Phe-Ser), showed a thixotropic and self-healing behavior. Understandably, ionizable side chains revealed a pH dependency of the gel-sol transition. For instance, cyclo(Phe-Glu) formed stable hydrogels at pH 6, while cyclo(Phe-Lys) at pH 8–11. The blended hydrogel obtained mixing these two CDPs showed remarkably wider pH stability from 2 to 11. The gel-to-sol transition temperature was dependent on the pH value, with a maximum corresponding to 79 °C at pH 2, and a minimum corresponding to 27 °C at pH 7 [103].

**Figure 4:**
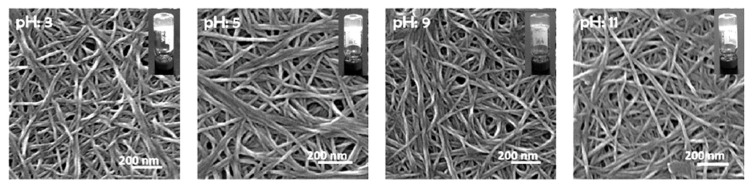
SEM images and photographs (insets) of the gels formed by cyclo(Phe-Leu) at various pH values. Adapted with permission from [101], copyright © 2019, Elsevier.

Cyclo(Phe-Leu) is a strong hydrogelator with self-healing ability and rheological stability in the temperature range corresponding to 20–60 °C [101]. The stability against heating highlighted the different behavior of CDPs relative to linear short peptides, whose gels remained stable at analogous conditions. Temperature changes can sometimes be accompanied by crystallization, especially if gels are a metastable phase. Cyclo(Phe-Leu) is not ionizable, and thus it showed a gelling ability through a wide range of pH values, from 3 to 11 (Figure 4). Another interesting feature of this compound was its demonstrated resistance against protease-catalyzed hydrolysis [101]. It is apparent that CDPs can be a good choice to make hydrogels suitable for medical applications, and offer various advantages over the linear-peptide analogs.

### 3.2. Gels from Protected-Dipeptide Derived CDPs

As can be seen from Table 1, the vast majority of reported CDP gelators based on unprotected dipeptides features hydrophobic amino acids. To further expand the chemical diversity of the building blocks and attain CDP hydrogelators also from hydrophilic amino acids, a popular strategy employs the use of hydrophobic protecting groups. These include Fmoc (fluorenylmethyloxycarbonyl) [107,110,111,112], O*^t^*Bu (*tert*-butoxy) [113,114], Boc [111], and, generally, alkyl-chain derivatizations [106,115], for instance through formation of amide bonds with lipophilic carboxylic acids, as described more in detail below.

#### 3.2.1. Protected CDPs with Glu or Asp Acidic Groups

Cyclo(Glu-Glu) is a hydrophilic CDP, featuring two ionizable carboxylic groups of the side chains of the glutamic acid residues. These functional groups were reacted with Fmoc-Cl as a protection strategy. In particular, the Fmoc mono-substituted CDP revealed an interesting gelation ability [110]. Without the Fmoc protecting group, the CDP was too hydrophilic to yield hydrogels. Instead, with the Fmoc mono-substitution, it gained ability as superhydrogelator, superorganogelator in aromatic and chlorinated solvents, and also as organogelator in alcohols. The racemate formed hydrogels with a slightly higher concentration (0.6 wt%). XRD data showed a “pseudoracemate” non-crystalline self-assembly, and this system showed a faster thixotropic recovery time than the enantio-pure analogs. The recovery time was also tunable with the concentration (using 0.6 to 1.2 wt%) from 4 to 10 min [110].

**Figure 5:**
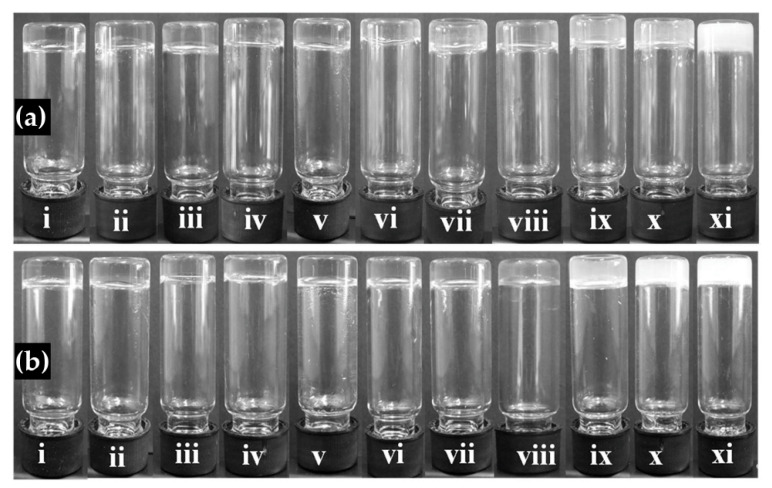
Photographs of gels formed by (**a**) cyclo(Tyr-Glu(O*^t^*Bu)) and (**b**) cyclo(Phe-Glu(O*^t^*Bu)) in various solvents: (i) toluene, (ii) chloroform, (iii) ethyl acetate, (iv) dichloromethane, (v) 1,2-dichlorbenzene, (vi) *tert*-butanol, (vii) *sec*-butanol, (viii) acetonitrile, (ix) 50% glycerol, (x) water, (xi) phosphate buffered saline (PBS, 10 mM, pH 7.4). Reproduced with permission from [113], copyright © 2017 American Chemical Society.

A series of four CDPs with Glu and Asp were protected with O*^t^*Bu and studied for the influence of the length of the side chain of Glu and Asp on their gelation ability [113]. The four compounds were thus cyclo(Tyr-Glu(O*^t^*Bu)), cyclo(Phe-Glu(O*^t^*Bu)), cyclo(Phe-Asp(O*^t^*Bu)), and cyclo(Leu-Glu(O*^t^*Bu)). The first two were compared to understand the role, if any, of the *para*-hydroxyl substitution on the aromatic side chain. Both were found to be super hydro- and organo-gelators in many solvents (Figure 5), therefore it seemed that the OH group of Tyr was not involved in the main driving-force interactions of the self-assembly process. The latter two were compared to elucidate the effect, if any, of the differing number by one carbon atom in the side chain. This small difference was sufficient to lead to two completely divergent supramolecular behaviors. In particular, the CDP featuring Glu was a super hydro- and organo-gelator, whilst that featuring Asp was almost insoluble in all the solvents, with organogels being formed only in 1,2-dichlorobenzene, and at high concentration (4.6 wt%), relative to the Glu-analogue in the same solvent (0.1 wt%). This latter CDP demonstrated to be a hydro- and organo-gelator, although it required higher concentrations to gel, relative to the aromatic analogs cyclo(Tyr-Glu(O*^t^*Bu)) and cyclo(Phe-Glu(O*^t^*Bu)), presumably due to the loss of the possibility to engage in π-π stacking and CH-π intermolecular interactions [113].

Cyclo(Glu-Glu) is functionalizable as a symmetric or asymmetric compound, thanks to the presence of two carboxylic groups in the side chains of the symmetric scaffold. A series of cyclo(Glu-Glu) derivatives were studied to understand the difference in supramolecular behavior between symmetric and asymmetric building blocks [114]. Tyramine, phenylalanine, tyrosine, and O*^t^*Bu-protection were the four variable structural components of this study; tyramine was linked to the side chain of Glu through the amino group, thus forming an amide bond. Phe or Tyr instead was derivatized through the α-amino group. O*^t^*Bu was added to the C-terminal carboxylic group of Phe or Tyr. All these building blocks were studied as asymmetric compounds (with functionalization occurring on only one of the Glu side chains) or symmetric (with both Glu being bonded to Tyramine, Phe, or Tyr). Tyramine demonstrated not to be a good choice, as both mono- and di-substituted CDPs did not gel. Without O*^t^*Bu on the carboxylic group of Phe or Tyr, no gels were formed either, the only exception being in acetone but only for two particular CDPs. The only hydrogelator was the Tyr-O*^t^*Bu mono-substituted CDP, while all the other compounds were good organogelators, although each one in different solvents. The presence of O*^t^*Bu seemed to play an important role in organogel formation in this series of analogs. As in the study described in the previous paragraph, mono-substitution at one Glu with a hydrophobic group enabled gelation. In particular, in this study, when cyclo(Glu-Glu) was mono-substituted with Phe or Tyr, it yielded a gelling agent as long as O*^t^*Bu was present on the free carboxylic group of Phe or Tyr [114].

#### 3.2.2. Protected CDPs with Lys Amino Groups

Lysine is another convenient amino acid to functionalize through the ε-amino group of the side chain. Protection with the Fmoc group increases the hydrophobicity and provides an aromatic character, which is useful to engage in non-covalent interactions for self-assembly. As an example, the amino acidic cyclo(Glu-Lys) was capped with O*^t^*Bu (Glu) and Fmoc (Lys) and compared against the unprotected analog [107]. With the capping of either one or both polar groups, all CDPs gained organogelation ability. When the O*^t^*Bu protecting group was present on the carboxylic moiety, the corresponding CDP became a superorganogelator for aromatic solvents, such as benzene, xylene, or toluene, with the possibility for the amine group to be either free or linked to Fmoc. When the carboxylic group was free and the Fmoc was installed on the amine, the CDP became a hydrogelator and an organogelator for chlorinated solvents [107].

Besides Fmoc, Boc is another convenient protecting group for the Lys ε-amine. A similar study on the influence of Boc and Fmoc groups showed that cyclo(Lys-Lys) did not form hydrogels, while the corresponding Fmoc and Boc derivatives were organogelators, although none of these compounds showed a minimum gelling concentration <1 wt% [111]. Another study showed the importance of the Fmoc-protecting group in the organogelation of cyclo(Lys-Lys) derivatives. The CDPs were functionalized with cysteine on both Lys-side chains through amide bonding, with the α-amino group of Cys being either Fmoc-protected or free. Only the derivatives with the Fmoc group were able to yield organogels, while thiols could be either in the reduced form or oxidized in a bicycle [112].

Alkyl chains provide another strategy to increase the hydrophobicity of polar side chains of CDPs. Lysine could be acylated with anhydrides or coupled with an aliphatic carboxylic acid through an amide bond. A study of these kinds of derivatives, using cyclo(Tyr-Lys) as CDP scaffold, showed the change in hydrophobicity was related to the different gelling behavior [106]. In particular, linear carboxylic acids with an aliphatic chain composed of 1, 2, 3, 5, 7, 11, or 17 carbon atoms were chosen as acyl functional groups. Low-molecular-weight acids (with 1–4 carbon-atom chains) rendered CDPs hydrogelators, with the lighter compound being also an organogelator for polar solvents, such as dimethylformamide, DMSO, and piperidine. The use of alkyl chains composed of 2–17 carbon atoms provided organogelation ability in alcohols [106].

## 4. Applications

**Figure 6:**
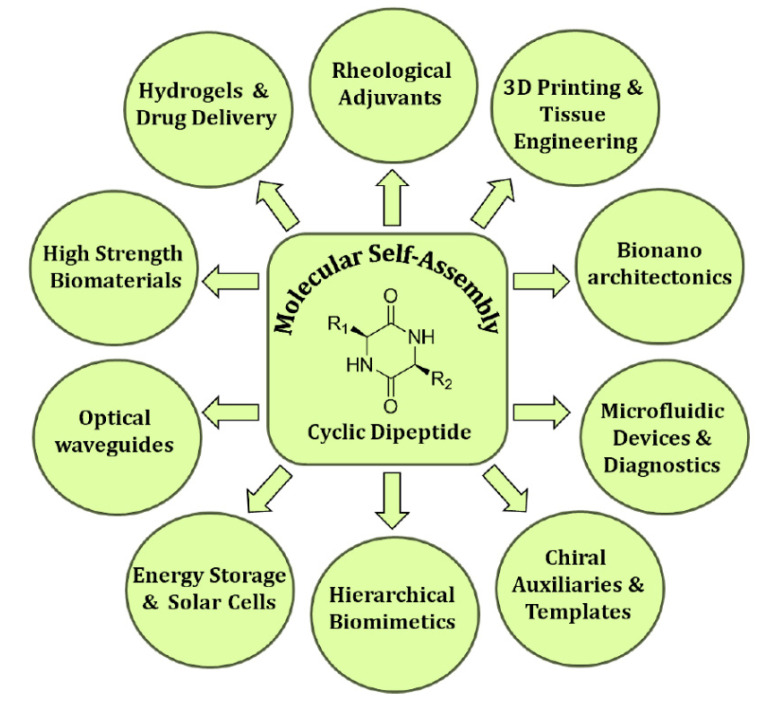
Various applications are envisaged for self-assembled CDPs. Reproduced with permission from [27], © 2017 Wiley-VCH Verlag GmbH & Co. KGaA, Weinheim.

Self-assembled gels are attractive systems to design innovative materials. As discussed in this review, CDPs can be biocompatible, cheap, and easy to synthesize, and natural CDPs provide a great source of inspiration to develop green solutions for many of the challenges of our century. As CDPs are a popular topic, recent reviews already exist on CDP applications in medicine [39] and optoelectronics [40]. A comprehensive review of CDP uses (Figure 6) was published in 2017 [27], therefore here we will focus on the latest advancements in the field.

One research area that is gaining momentum is bioinspired enzyme mimicry through the design of supramolecular catalysts based on minimalistic peptides [116] and their derivatives, CDPs included. For instance, His and Cys residues are catalytically active residues of various hydrolases. Cyclo(Phe-His) and cyclo(Phe-Cys) successfully self-assembled into a hydrogel for esterase mimicry [41]. Cyclo(Phe-Leu) formed a hydrogel in the presence of tetramethylpyridylporphyrin iron complex (Fe(III)-TMPyP) as a catalyst for peroxidase reactions [82]. The hydrogel not only provided a scaffold but also enhanced the catalyst acceleration of pyrogallol oxidation, relative to the free catalyst in solution [82].

**Scheme 4:**
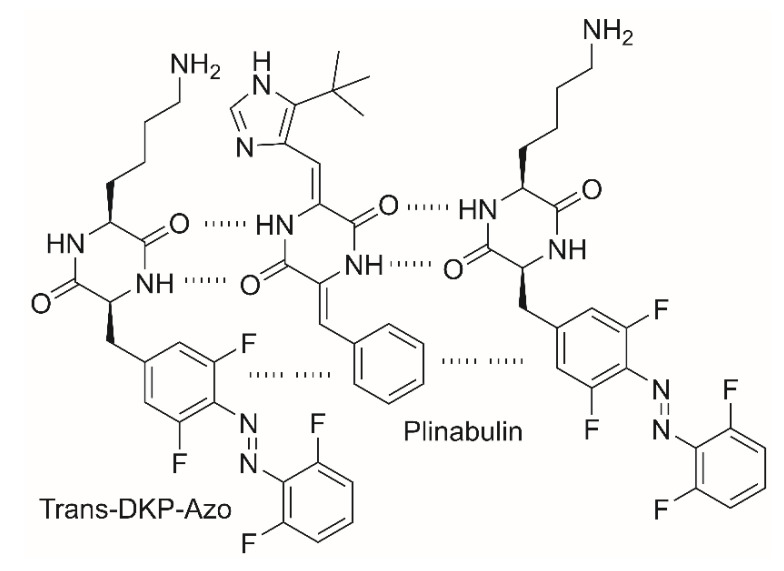
Chemical structures of the photo-responsive trans isomer of an azobenzene-derivative of a DKP (Trans-DKP-Azo) that self-assembles into a hydrogel and can be used to carry the anticancer drug plinabulin (center) through non-covalent interactions [117].

Another area that is very promising for its innovative potential consists of the development of smart materials, which respond to a variety of stimuli, with light being particularly attractive for spatio-temporal control. For instance, a biocompatible non-natural CDP equipped with an azobenzene (Scheme 4) provided an interesting hydrogelator that responded to a light switch through sol-gel transitions [117]. In particular, green-light irradiation promoted *trans*-to-*cis* isomerization, which could be reversed upon appropriate change of the light wavelength. Since only the *trans* isomer was a gelling agent, it was thus possible to switch from the gel to a solution upon green-light irradiation. The compound was studied to carry a possible anticancer drug into a living system and then release it with an external-light stimulus [117].

Finally, organic electronics is one of the most researched areas as finding new technologies in this field could translate into great innovations in the electronic market. Recent advancements include organic micro-devices that can be printed avoiding traditional silicon-based components, although at present this is sustainable only in batch [118]. For large-scale production, the key is finding a few versatile organic materials to reduce the number of components needed for the printing process and to optimize the number of reagents needed to print a complex circuit. The high stability provided by CDPs, together with their easy low-cost production on a large scale, indeed makes them attractive to develop organic electronics [40]. For example, cyclo(Tyr-Trp) is a hydrogelator with good resistance to harsh environments. This compound was tested at different pH and temperature values, as well as in the presence of charged biopolymers, and the noticeable resistance suggested the potential for electronic applications. Cyclic voltammetry studies showed a supercapacitor behavior and good electrochemical stability, thus this material could be a good candidate for an organic supercapacitor component [105].

## 5. Conclusions

The area of self-assembling minimalistic systems composed of di- and tri-peptides has attracted great interest in the last decade, showing promising potential for a variety of applications. Recently, we have witnessed a renaissance of their cyclic derivatives, CDPs, in virtue of their higher stability against physico-chemical harsh conditions as well as biological degradation. The rigid structure provided by the CDP ring is particularly attractive to impart various bioactivities, to allow to cross the blood-brain barrier and reach otherwise difficult pathological targets to treat, and also to favor self-assembly into hydro- and organo-gels. As new bioactive CDPs continue to be identified from natural sources, and their synthesis can be easily attained also through the development of biotechnological and microwave-assisted green methods, it appears evident that the field is well-set to keep growing and bring further innovation in all the areas where greener alternatives are highly sought after.

## Figures and Tables

**Table 1:** Gelling CDPs obtained from unprotected dipeptides.

CDP Sequence	Gel Type	Reference
Cyclo(Ala-Gly)	Organogel	[100]
Cyclo(Val-Gly)	Organogel	[100]
Cyclo(Leu-Gly)	Organogel	[100]
Cyclo(Leu-Val)	Organogel	[100]
Cyclo(Leu-Ala)	Organogel	[100]
Cyclo(Leu-Leu)	Organogel	[100]
Cyclo(Phe-Gly)	Organogel	[100]
Cyclo(Phe-Gly)	Hydrogel	[41]
Cyclo(Phe-Val)	Hydrogel	[86]
Cyclo(Phe-Leu)	Hydrogel	[82,101]
Cyclo(Phe-Leu)	Organogel	[100,102]
Cyclo(Phe-Phe)	Hydrogel	[86]
Cyclo(Phe-Phe)	Organogel	[100]
Cyclo(Phe-Cys)	Hydrogel	[103]
Cyclo(Phe-Ser)	Hydrogel	[103]
Cyclo(Phe-Glu)	Hydrogel	[103]
Cyclo(Phe-His)	Hydrogel	[103]
Cyclo(Phe-Lys)	Hydrogel	[103]
Cyclo(Trp-Trp)	Organogel	[104]
Cyclo(Trp-Tyr)	Hydrogel	[105]
Cyclo(Tyr-Tyr)	Hydrogel	[104]
Cyclo(Tyr-Tyr)	Organogel	[104]
Cyclo(Tyr-Lys)	Hydrogel	[106]
Cyclo(Tyr-Lys)	Organogel	[106]
Cyclo(Lys-Glu)	Organogel	[107]
